# The Roles of Nanoparticles in Stem Cell-Based Therapy for Cardiovascular Disease

**DOI:** 10.3389/fbioe.2020.00947

**Published:** 2020-08-14

**Authors:** Yuting Sun, Yuexin Lu, Li Yin, Zhenjie Liu

**Affiliations:** ^1^Department of Surgical Oncology, The Second Affiliated Hospital, Zhejiang University School of Medicine, Hangzhou, China; ^2^Department of Vascular Surgery, The Second Affiliated Hospital, Zhejiang University School of Medicine, Hangzhou, China

**Keywords:** nanoparticles, stem cell, cardiac disease, peripheral vascular disease, regeneration

## Abstract

Cardiovascular disease (CVD) is currently one of the primary causes of mortality and morbidity worldwide. Nanoparticles (NPs) are playing increasingly important roles in regulating stem cell behavior because of their special features, including shape, size, aspect ratio, surface charge, and surface area. In terms of cardiac disease, NPs can facilitate gene delivery in stem cells, track the stem cells *in vivo* for long-term monitoring, and enhance retention after their transplantation. The advantages of applying NPs in peripheral vascular disease treatments include facilitating stem cell therapy, mimicking the extracellular matrix environment, and utilizing a safe non-viral gene delivery tool. However, the main limitation of NPs is toxicity, which is related to their size, shape, aspect ratio, and surface charge. Currently, there have been many animal models proving NPs’ potential in treating CVD, but no extensive applications of stem-cell therapy using NPs are available in clinical practice. In conclusion, NPs might have significant potential uses in clinical trials of CVD in the future, thereby meeting the changing needs of individual patients worldwide.

## Introduction

Cardiovascular disease (CVD) is one of the primary causes of mortality and morbidity around the world (GDB 2017 DALYs and HALE Collaborators, 2018). According to the latest data, ischemic heart disease remains the primary cause of death among various CVD (Prajnamitra et al., 2019). Although the current pharmacological treatments and surgical procedures have improved the survival rate of the patients with cardiac disease, none of these therapies mentioned above can replace the cardiac tissue lost after infarction (Calin et al., 2013). Peripheral vascular disease (PVD), which is characterized by the narrowing of the peripheral vasculature, has a significant unfavorable impact on the health of patients (Bartolo et al., 2019). However, traditional strategies have limited efficacy in treating PVD (Tu et al., 2015; Hedhli et al., 2017). Therefore, regenerative medicine is urgently needed to achieve the functional recovery of damaged tissues. Stem cell therapy is a potential alternative approach to achieve therapeutic angiogenesis with several unique advantages over growth factor therapy or gene therapy. However, the efficacy of stem cells to treat CVD remains controversial. A meta-analysis of 52 preclinical animal studies of MSC therapy for ischemic heart disease showed that it is safe and associated with moderate (∼7.5%), but significant improvements in left ventricular ejection fraction, but the effect of cell therapy on left ventricular ejection fraction decreased slightly after 8-week follow-up (van der Spoel et al., 2011).

Nanoparticles and structures have been used by humans in the fourth century AD by the Roman, which demonstrated one of the most interesting examples of nanotechnology (Bayda et al., 2019). The use of nanoparticles (NPs) can help overcome some of the obstacles as mentioned above and augment the benefits of cell therapy through delivering genes to stem cells, enhancing stem cell retention, facilitating the proangiogenic effect of stem cells and mimicking the extracellular environment. This review mainly focuses on the types, physical characteristics, adverse effects of NPs, and the mechanisms by which NPs have improved stem cell-based treatment strategies for cardiovascular regeneration in recent years. Furthermore, we also aim to illustrate the challenges that we face in applying nanotechnology for cardiovascular regeneration and its prospects for the future.

## Stem Cell Types

In terms of heart disease, mesenchymal stem cells (MSCs) constitute a potential option for cell-based therapy to treat cardiac disease. The results of the RIMECARD clinical trial showed that human-derived MSCs could improve the cardiac function of patients with myocardial infarction (MI) (Hare et al., 2009). The POSEIDON-DCM trial showed that MSCs could reduce fibrosis in scarred tissues (Hare et al., 2017). Additionally, a clinical trial aiming to investigate the safety and efficacy of the intramyocardial implantation of allogeneic MSCs in patients with end-stage ischemic cardiomyopathy has been initiated, but the results have not yet been reported. Human-derived induced pluripotent stem cells (hiPSC) constitute another stem cell type that might be suitable for stem cell-based therapy, and two recent studies demonstrated that human embryonic stem cell-derived cardiomyocytes enhance cardiac function in macaque monkeys with large MI areas (Shiba et al., 2016; Liu et al., 2018). However, no clinical trials have been demonstrated their effects on patients with cardiac disease. In terms of vascular disease, MSCs play a role in vessel regeneration that is as important as their role in cardiac disease. A pilot study demonstrated that adipose-derived MSCs injections might be a safe alternative to achieve therapeutic angiogenesis in patients with critical limb ischemia (a severe manifestation of PVD). However, the sample size was small, and the results are, therefore, insufficient (Lee et al., 2012). A phase II clinical trial shows that the use of MSCs combining with endothelial progenitor cells (EPCs) therapy is safe and effective for increasing blood flow in the ischemic legs of patients with limb ischemia (Lasala et al., 2012), also, EPCs have been applied in bovine models and murine models of peripheral arterial disease and can be isolated from hiPSC (Asahara et al., 1997; Boyer et al., 2000; Samuel et al., 2013; Peters, 2018).

However, several limitations of treating CVD with stem cells exist, including the low survival rate of implanted cells (Yun and Lee, 2019), the rapid apoptosis of the transplanted cells because of exposure to a hypoxia environment (Tang et al., 2005) and the poor survival of the transplanted cells caused by the inflammatory and proapoptotic environment in ischemic tissue (Zhang et al., 2001; Toma et al., 2002). Many studied have shown stem-cell therapy with NPs.

## NPs Structure and Types

### The Definition of NPs

The published recommendation for the definition of nanomaterials refers to a nanomaterial as a natural, incidental or manufactured material consisting of particles in an unbound state, aggregate or agglomerate consisting, and indicates that 50% or more of the particles should be in a size distribution range from 1 to 100 nm based on one or more external dimensions (Piperigkou et al., 2016). Because of their unique physical and chemical properties, NPs have played an increasingly important role in determining and regulating stem cell behavior, including tracing the fate and distribution of stem cells *in vivo*, inducing directed differentiation of stem cells, ascertaining the origin of stem cell diseases, stimulating the paracrine behavior of stem cells and regulating the microenvironment of tissues around stem cells (La Francesca, 2012; Le et al., 2017). Various types of NPs exist, including metal NPs, SPIONs, SNPs, carbon NPs. In this section, we will review the common types of NPs used in cardiovascular regeneration.

### Physicochemical Properties of NPs

#### Particle Size and Surface Area

Particle size and surface area play significant roles in the interaction between materials and biological systems (Talkar et al., 2018), for example, how a system responds to absorb, distribute, and eliminate the materials (Powers et al., 2007). According to the size of the NPs, the uptake modes can be categorized as phagocytosis and pinocytosis. Large particles are mainly absorbed through phagocytosis, while small particles can be absorbed through either phagocytosis or pinocytosis, with the larger particles internalized by the cells with the most enormous phagocytosis capacity (Aillon et al., 2009).

#### Particle Shape and Aspect Ratio

The processes of endocytosis and phagocytosis are affected by the shape characteristics of the material. Spherical particles are more natural to endocytose than particles of any other shape. Non-spherical nanomaterials are more likely to flow through capillaries, causing other biological consequences (Lee et al., 2007; Verma and Stellacci, 2010; Kim et al., 2012).

#### Surface Charge

The surface charge of NPs has a significant influence on the biological system, and the surface charge on the particles determines many of their interactions (Georgieva et al., 2011). The mammalian cell membrane has a negatively charged surface. Therefore, cationic particles are more likely than negative or neutral particles to interact with cells.

#### Other Characteristics

The crystalline structure, aggregation potential, and surface coating of the NPs can also affect their biocompatibility and toxicity (Pietroiusti et al., 2011). Concentration may be the main factor contributing to the toxicity of large particles (Gatoo et al., 2014; Talkar et al., 2018).

### The Most Commonly Used Types of NPs in CVD

#### SPIONs

In magnetic resonance imaging (MRI), SPIONs exhibit magnetism in the region exposed to the magnetic field, and when the external magnetic field is removed, the magnetism of the SPIONs also disappears. SPIONs, as T2 magnetic contrast agents, mainly affect MRI R2 relaxation, shorten the T2 time, and weaken the weighted T2 signal. They are characterized by having a size in the nanometer range, with intense penetration, and a relaxation rate of 7∼10-fold higher than that of Gd3+ at the same concentration, which enables MRI at very low concentrations, revealing a region of signal reduction that forms a contrast with the signals of the surrounding tissues (Urdzíková et al., 2006). The magnetic properties of SPIONs, which are influenced by external magnetic fields, are used as cell markers (Cromer Berman et al., 2011). Among cell marker SPIONs, magnetic Fe_3_O_4_ has the most application prospects. SPIONs have the characteristics of superparamagnetism, low toxicity, excellent biocompatibility, and directional movement under an external magnetic field (Bull et al., 2014). These NPs have broad application prospects as cell markers, guide molecules, and probes for monitoring the therapeutic effect of stem cells on acute MI. Leveraging their magnetic properties, SPIONs are used in MRI to tag and, effectively trace cells to determine cell localization. SPIONs with particle sizes of 1∼100 nm are not quickly engulfed by the reticuloendothelial system. SPIONs have a positive surface charge and often require surface modification because SPIONs without surface modification are unstable and tend to polymerize in the moist environments.

#### Quantum Dots (QDs)

Quantum dots are important nanoscale, low-dimensional semiconductor materials that, in three dimensions, do not exceed twice the exciton Bohr radius of the corresponding semiconductor material (Lei et al., 2008). QDs are generally spherical, and their diameters are usually between 2 and 20 nm. Because of their unique optical properties (broad excitation and narrow emission spectra), they emit brighter and particularly stable fluorescence (Michalet et al., 2005). A previous study presented an immunomagnetic assay based on functionalized magnetic beads and detachable QDs for the separation and quantication of soluble CD40 Ligand (CD40 Ligand, also known as tumor necrosis factor associated activation protein, is related to atherosclerosis) from solution (Park et al., 2013). Recently, a study firstly reported that 0D titanium carbide MXene QDs could be incorporated into a chitosan-based hydrogel to create a 3D platform with enhanced physicochemical properties for stem cell delivery and tissue repair (Rafieerad et al., 2019). Furthermore, another study demonstrated that selenium QDs can prevent endothelial dysfunction and reduced the size of atherosclerotic plaque in aortic arteries (Zhu et al., 2019).

#### SNPs

SNPs are ultrafine with a size range from 1 to 100 nm. Therefore, they have many unique optical properties in ultraviolet light, which can enhance the aging resistance, strength, and chemical resistance of other materials (Tang et al., 2012). SNPs can be divided into two categories: solid SNPs and mesoporous SNPs, both of which have the potential uses in stem cell therapy, including stem cell differentiation, imaging, and tracking (Tang and Cheng, 2013; Patel and Lee, 2015). Additionally, they have unique biological adaptability and show morphological adjustability plasticity (Slowing et al., 2008).

#### Metal NPs

Among various metal NPs, the most commonly used metal-based NPs consist of silver, gold, copper, iron, and zinc (Iravani et al., 2014; Kandi and Kandi, 2015). Nanomaterials, including gold NPs (AuNPs), demonstrated excellent biocompatibility (Hung et al., 2009b, 2012) and possessed unique optical and surface plasmon resonance properties (Singh et al., 2018). Numerous plants and microorganisms have the potential to produce AuNPs (Alcor et al., 2009; Singh et al., 2016, 2017; Sánchez-López et al., 2020). These biologically synthesized AuNPs have become potential options for use as biosensors and in immunoassays, targeting drug delivery, photoimaging, photothermal therapy, and photodynamic therapy (Singh et al., 2018). The functionalization of AuNPs may enable cardiomyocyte to differentiation to hiPSC (Jung et al., 2012; Patel et al., 2014). Additionally, AuNPs promoted the exosome secretion from MSCs and inhibited autophagy flux in MSCs (Arslan et al., 2013). Exosomes secreted by MSCs play an important role in ischemic disease and the inflammatory response. Promoting exosome secretion is of great significance for the use of MSCs in the treatment of MI, lower limb ischemia, cerebral infarction, and other diseases (Arslan et al., 2013; Chen et al., 2015).

#### Solid Lipid NPs (SLN)

Solid lipid NPs, with a size range of 50–1000 nm, is composed of a solid lipid core surrounded by a layer of surfactants in aqueous dispersion, with multiple potential combinations of lipids and surfactants (Chauhan et al., 2016). SLN made their first appearance almost 25 years ago and are one of the newest members of the lipid-based nanocarriers family (Gasco, 2007; Satterlee and Huang, 2016). SLN among the most effective carriers for hydrophilic and hydrophobic drugs owing to their inclusion of cationic lipids, which provide a positive surface potential that favors binding to nucleic acids; and SLNs can be used for gene delivery, which provides a positive surface potential that favors binding to the nucleic acids like DNA, siRNA, miRNA (Naseri et al., 2015; Botto et al., 2017).

#### Other Types

Other types of NPs include micelles (Yohan and Chithrani, 2014), lipid-calcium-phosphate nanoparticle (Satterlee and Huang, 2016), microalgae-nanoparticle (Déniel et al., 2019), protein-based NPs (Tarhini et al., 2017), liposomes and polymeric NPs (Tapeinos et al., 2017). The advantages and disadvantages of different types of NPs are shown in Table 1.

**TABLE 1:** The advantage and disadvantages of different types of NPs.

NPs types	Advantages	Disadvantages	Application in CVD
SPIONs	Low toxicity, excellent biocompatibility, and directional movement under the external magnetic field	SPIONs without surface modification are unstable and tend to polymerize in the liquid environment; remain in the body and produce images that misdirect the tracer and overestimate the stem cells’ survival	Cell markers, guide, and monitor the therapeutic effect of stem cells on acute myocardial infarction
QDs	Brighter and more stable fluorescence; resistance to chemical and biological degradation	The high toxicity of QDs when they are using in vivo systems	Used for contrasting of blood and lymph vessels; cell labeling; tumor diagnosis and therapy
SNPs	Optical properties against ultraviolet light; unique biological adaptability and morphological adjustability	15 nm diameter particles have been reported to trigger more cytotoxicity than 100 nm diameter particles in endothelial cells	Have the potential in stem cell therapy including stem cell differentiation, imaging, and tracking
Metal NPs	Excellent biocompatibility; unique optical and SPR properties	Severe side effects due to its oxidation state	AuNPs could potentialize the cardiomyocyte differentiation of human pluripotent stem cells and promote exosome secretion of MSCs and inhibit autophagy flow of MSCs

*AuNPs, gold nanoparticles; CVD, cardiovascular disease; MSCs, mesenchymal stem cells; NPs, nanoparticles; QDs, Quantum dots; SPIONs, superparamagnetic iron oxide nanoparticles; SNPs, silica nanoparticles; SPR, surface plasmon resonance.*

## Stem-Based Therapy With NPs in CVD

### NPs’ Application in Cardiac Regeneration

#### NPs Deliver Genes to Stem Cells

Previous studies have shown that genetic engineering can be used to facilitate stem cell therapy for heart disease (Hodgkinson et al., 2010; Marbán and Malliaras, 2012) by introducing therapeutic genes (proangiogenic and antiapoptotic genes) into engineered stem cells through gene vectors to prolong their survival and enhance their paracrine secretion (Deuse et al., 2009; Tang et al., 2009). Due to the immune responses and low gene volume, traditional vectors have limited applications. NP-based genes with unique biocompatibility may have higher gene delivery efficacy when transferred to stem cells, increasing cell survival and differentiation in the ischemic myocardium. Several types of nanostructured gene vectors exist. Liposomes can prevent genes from being degraded or binding non-specifically (Pack et al., 2005; Zhang et al., 2008); polymers can increase the efficiency, reduce the cytotoxicity and improve targeting specificity (Cui et al., 2015); inorganic NPs have simple fabrication requirements, inducing low cytotoxicity (Kim and Hyeon, 2014) and can be used with blended vectors. The mechanisms of their internalization are known as endocytic pathways, including clathrin-mediated endocytosis, caveolae-mediated endocytosis, and micropinocytosis and phagocytosis (Xiang et al., 2012; Calin et al., 2013). A study used chitosan-alginate NPs to deliver placental growth factors in a targeted manner, which could achieve the goal of continuously releasing placental growth factors and improving cardiac function at the site of acute MI (Binsalamah et al., 2011). Another study transfected MSCs with molecularly organic-inorganic hybrid hollow mesoporous organosilica NPs with surface conjugated polyethyleneimine loaded with the hepatocyte growth factor gene and found that the paracrine activity of the hepatocyte growth factor-transfected MSCs was enhanced, which reduced the myocardial cell apoptosis and promoted angiogenesis in the rat model of MI (Zhu et al., 2016). Several studies have shown the application of different types of NPs in gene delivery (Table 2).

**TABLE 2:** NPs applications for gene delivery.

NPs	Stem cells	Gene	Model	Model
Chitosan-alginate NPs (Binsalamah, 2011)	–	PlGF	MI	Rat
Hpamam (Zhu et al., 2011)	SkMs	hVEGF-165	MI	Rat
Polyethyleneimine (Ye et al., 2011)	SkMs	pHRE- VEGF	MI	Rabbit
Bac-NP (Paul et al., 2012)	hADSCs	Ang-1	MI	Rat
PMSNs (Wenbin et al., 2015)	MSCs	CCR2-siRNA	MI	–
HMONs-PEI (Zhu et al., 2016)	BMMSCs	pHGF	MI	Rat

*Bac-NP, recombinant baculovirus complexed with a cell-penetrating transactivating transcriptional activator peptide/deoxyribonucleic acid nanoparticle; BMMSCs, bone marrow-derived mesenchymal stem cells; CCR2, C-C chemokine receptor 2; hADSCs, human adipose tissue-derived stem cells; HGF, hepatocyte growth factor; HMONs-PEI, molecularly organic-inorganic hybrid hollow mesoporous organosilica nanoparticles with surface conjugated polyethyleneimine; hPAMAM, hyperbranched polyamidoamine; Hvegf-165, human vascular endothelial growth factor-165; MI, myocardial infarction; MSCs, mesenchymal stem cells; NPs, nanoparticles; pHRE-VEGF, hypoxia-regulated vascular endothelial growth factor; PlGF, Placental growth factor; PMSNs, photoluminescent mesoporous silicon nanoparticles; SkMs, skeletal myoblasts.*

#### NPs Track the Stem Cells *in vivo*

As mentioned above, the fate, behavior, and survival of stem cells after transplantation *in vivo* remain unclear. Therefore, an efficient tool to monitor and track stem cells for long-term monitoring is necessary. SPIONs mark stem cells in three main ways: by attaching NPs to the cell surface through the internalization of NPs by the cells by through endocytosis, by receptor-mediated endocytosis, and by transfecting agent-mediated endocytosis (Frank et al., 2002). For *in vivo* experiments, the first approach has significant limitations, as the reticuloendothelial system recognizes and clears SPION-labeled cells (Suzuki et al., 2007; Nucci et al., 2015). However, through the internalization pathway, SPIONs can persist in the cytoplasm of stem cells where they have excellent biocompatibility. Currently, methods to enhance SPIONs transfer across membranes include increasing the electromagnetic fields to target SPIONs toward irradiated sites, ligand modifications on the surface of SPIONs to bind a receptor on the targeted cell membrane, ensuring specific SPIONs binding to the target cell, and promoting mononuclear-phagocytic cell phagocytosis of SPIONs, thus promoting passive transport (Lewin et al., 2000; Frank et al., 2002; Kraitchman et al., 2011). QDs have the potential for use in long-term monitoring in living cells, compared with traditional fluorescent probes (Ricles et al., 2011; Liu et al., 2019). Several studies have reported the feasibility of labeling stem cells through different modifications such as bioconjugated (Shah and Mao, 2011), electroporation (Sun et al., 2014), peptide delivery (Chang et al., 2008) and encapsulation and delivery by phospholipid micelles (Dubertret et al., 2002), all of which maintain the stability and safety of the label (Wang et al., 2015b). Silica dioxide NPs are applied as ultrasound contrast agents. They are usually combined with fluorescein, helium ions, or radionuclides to improve the imaging of the stem cells, thereby enabling stem cell tracking (Accomasso et al., 2012). Exosome-like silica, which has a unique morphology, provides a double-reflection interface that confers labeled stem cells with higher echogenicity and ultrasound sensitivity (Chen et al., 2017). In recent studies, different types of NPs have been applied in stem cell tracking for cardiac repair *in vivo* and *in vitro* (Table 3).

**TABLE 3:** NPs applications for stem cell tracking during cardiac repair.

NPs	Stem cells	Imaging modality	Model
SNPs (Fang Chen, 2020)	hMSCs	Ultrasound imaging	In vitro
Au@BSA@PLL (Ning et al., 2019)	hMSCs	CT	Rat
USPIO (Mathiasen et al., 2019)	MSCs	MRI	IHD patients
PANPs (Qin et al., 2018)	hESC-CMs	Photoacoustic imaging	Rat
Iron oxide NP (Skelton et al., 2016)	hESC-CPCs	MRI	Pig
PFCE-NPs (Constantinides et al., 2019)	CPCs	MRI	Rat
SiO_2_-NPs (Gallina et al., 2015)	hMSCs	Immunofluorescence analysis of tissue slices	Rat

*Au@BSA@PLL, gold nanoparticles (AuNPs) were synthesized on bovine serum albumin (BSA) via an *in situ* growth method and modified with a poly-L-lysine (PLL) layer; CPCs, cardiac progenitor cells; CT, computer tomography; hESC-CM, embryonic stem cell-derived cardiomyocytes; hMSC, human mesenchymal stem cells; IHD, ischemic heart disease; MRI, magnetic resonance imaging; NPs, nanoparticles; PANPs, photoacoustic nanoparticles; PFCE-NP, perfluorocarbon nanoparticles; SNPs, silica nanoparticles.*

#### NPs Enhance Stem Cell Retention After Transplantation

**FIGURE 1:**
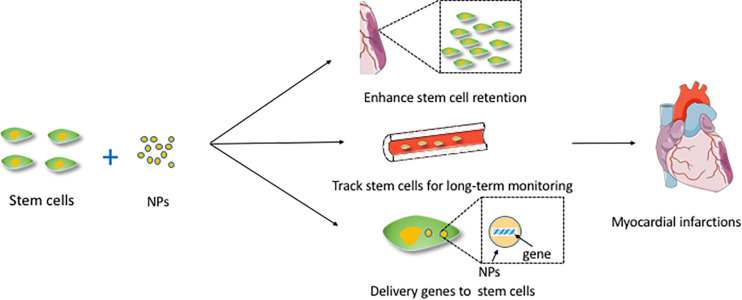
The mechanisms of NPs combined with stem cells and applied for cardiac regeneration. This figure sets an example of NPs used in myocardial infarctions. NPs, nanoparticles.

Different routes of stem cell administration include intracoronary (Vulliet et al., 2004; Liu et al., 2016; Vilahur et al., 2017), transendocadial method (Amado et al., 2005), systemic method (Barbash et al., 2003), and intravenous method (Kulandavelu et al., 2016). Extremely low cell retention after transplantation is the most critical barrier to the application of stem cell therapy for cardiac repair. As much as 1.5% of transplanted stem cells accumulated in the myocardium after only 2 h (Al Kindi et al., 2008). NPs may be a potential approach to address this problem. It has been demonstrated that NPs can enhance stem cell retention by providing a cardiac-specific extracellular matrix (ECM) environment (include impacts some signal pathway), affecting the focal adhesion complex of myocardial MSCs (Bejleri et al., 2018), promoting the interactions with cardiomyocytes and affecting lysosomal function in ischemic environments (Popara et al., 2018). Magnetic NPs seem to resolve this accumulation to some extent. This ameliorating effect may be associated with the magnetic field, and the retention of SPION-labeled stem cells may be significantly enhanced. The SPIONs can be applied not only for tracking stem cells but also for studying other aspects in a magnetic field. The advantage of magneto-electroporation in the delivery of cardiac stem cells is that it reduces the incubation time needed for NPs to label the cells markers (Calin et al., 2013). A study found that using SPION-labeled MSCs in the presence of a magnetic field might enhance cell homing of MSCs at the site of injury and contribute to the improvement of cardiac function and attenuation of injury after heart failure (Naseroleslami et al., 2018). The recruitment and leaching of Ly6Chigh mononuclear cells in the ischemic heart were associated with the signal transduction of the chemokine ligand 2/chemokine receptor 2 (CCR2), which was required to control pro-inflammatory monocytes and enhance the inflammatory microenvironment that adversely affects MSCs transplantation. A study applied photoluminescent porous SNPs and MSCs loaded with silencing CCR2 for the treatment of ischemic myocardial injury (Lu et al., 2015). The silencing CCR2 moiety targeted the expression of CCR2 in Ly6Chigh inflammatory monocytes, reducing the accumulation of these cells during infarction, thereby enhancing the efficacy of MSC transplantation and myocardial remodeling. Figure 1 shows the mechanisms of NPs combined with stem cells and applied for cardiac regeneration.

### NPs’ Application in Peripheral Vascular Regeneration

#### NPs Facilitate the Proangiogenic Effect of Stem Cells

The advantages of cell-based therapy with NPs have been proven in many practices. For example, MSCs have been demonstrated to augment collateral perfusion through paracrine mechanisms (Kinnaird et al., 2004). Compared with the use of proangiogenic factors alone, the efficiency of induced angiogenesis was more stable and lasted longer with a combination of stem cells and NPs. Recently, a study proposed that tethering the adipose-derived stem cells (ADSCs) surface with NPs releasing tumor necrosis factor α, also named nanostimulators, stimulated cellular secretory activity *in situ*, and the results showed that ADSCs with tethered nanostimulators promoted vascularization in a 3D microvascular chip and enhanced the recovery of cell perfusion, animal walking and muscle mass in murine ischemic hind limbs compared to the effect of untreated ADSCs (Leong et al., 2020). Another study has proven that human MSCs incubated with magnetic NP-containing liposomes showed increased expression of vascular endothelial growth factor (VEGF) and a reduced apoptosis rate in unilateral hind limb ischemic animal models compared to these effects in the control group (Ishii et al., 2011). Similarly, a study showed that chitosan oligosaccharide/heparin NPs had a high cytokine-loading capacity and allowed cytokines to maintain stable bioactivity longer in an environment at physiological pH *in vitro* (Wang et al., 2015a). In recent years, metal nanomaterials have offered the potential to improve the efficiency of vascular regeneration. A study in 2004 firstly proved that AuNPs have angiogenesis properties. The plausible mechanism could be that controlled reactive oxygen species generation and consequently reduced redox signaling (Nethi et al., 2014). A similar mechanism was proven in the treatment of hepatic ischemia-reperfusion using ceria NPs (Ni et al., 2019). Later, another study indicated that VEGF on fibronectin-incorporated AuNPs promoted MSCs migration through the endothelial oxide synthase/metalloproteinase signaling pathway, which promoted MSC proliferation and increased the biocompatibility of the particle (Chen et al., 2018). Table 4 shows NPs applications in promoting stem cells to secrete factors related to angiogenesis.

**TABLE 4:** NPs applications for promoting stem cells to secrete factors related to angiogenesis.

NPs	Stem cells	Relative factors	Model
Magnetic NPs (Ishii et al., 2011)	hMSCs	VEGF	Nude mouse
AuNPs (Chen et al., 2018)	MSCs	CD31, α-SMA	*In vitro*
Cerium oxide NPs (Das et al., 2012)	HUVECs	VEGF, HIF-1α	*In vitro*
ZnO NPs (Augustine et al., 2014; Ahtzaz et al., 2017)	HUVECs	VEGF, FGF2	*In vitro*

*α-SMA, alpha-smooth muscle actin; AuNPs, gold nanoparticles; FGF2, fibroblast growth factor 2; hMSCs, human mesenchymal stem cells; HIF-1α, hypoxia-induced factor 1 alpha; HUVEC, human umbilical vein endothelial cells; NPs, nanoparticles; MSCs, human mesenchymal stem cells; VEGF, vascular endothelial growth factor; ZnO NPs, zinc oxide nanoparticles.*

#### NPs Mimic the ECM Environment

Extracellular matrix is a complex environment that plays a role in maintaining cell and tissue structure and function (Rosso et al., 2004), and its components and three-dimensional ultrastructure transmit specific signals to cells (Sorokin, 2010). Nanomaterials used for tissue engineering have been proposed to mimic ECM. A previous experiment described the development of hyaluronan oligomers-loaded poly NPs used for the targeted, controlled, and sustained delivery of hyaluronan oligomers for the elastogenic induction of aneurysms in aortic smooth muscle cells in rat models (Sylvester et al., 2013). The ECM is a polymer-based microenvironment that serves as a pool for signaling molecules (Afratis et al., 2012; Mecham, 2012). EPCs are ideal for use in inducing primary endothelial cells for vascular tissue engineering (Hung et al., 2009a). EPCs migration and homing to ischemic tissues are mediated by cell adhesion molecules and chemokines (Landmesser et al., 2004). Stromal-derived factor 1α (SDF-1α) and its receptor C-X-C motif chemokine receptor (CXCR4) are essential molecules that direct cell migration. A study has demonstrated that collagen combined with AuNPs nanocomposites may accelerate the proliferation and migration of MSCs and stimulate endothelial cell differentiation to facilitate vascular regeneration (Hung et al., 2014a). Another study showed that EPC-seeded mixtures of polyurethane (PU, which might be produced to individual match the microenvironment of vascular tissue precisely) and Au-NPs differentiate into endothelial cells *in vitro*, and the enhanced maturation of the EPCs with the nanocomposites of PU-Au-NPs was more remarkable, probably because of the SDF-1α/CXCR4 signaling pathway *in vitro*. Furthermore, other experiments showed that EPCs seeded on PU-Au-coated catheters effectively reduced thrombosis by differentiating into endothelial cells *in vivo* (Hung et al., 2014b). However, no clinical trials have explored whether this kind of therapy would have a favorable effect on PVD patients.

#### NPs Act as a Non-viral Gene Delivery Tool

**FIGURE 2:**
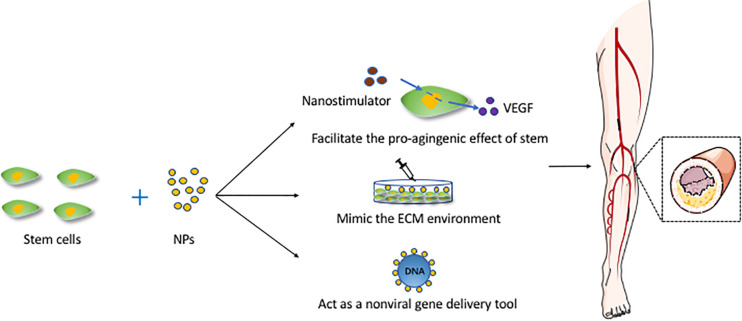
The mechanisms of NPs combined with stem cells and applied for vascular regeneration. This figure sets an example of NPs used in artery atherosclerosis. ECM, extracellular matrix; NPs, nanoparticles; VEGF, vascular endothelial growth factor.

Adipose-derived stromal cell populations contain MSCs (Zuk et al., 2001), which shows the potential to release VEGF, and upregulating VEGF has been proved to enhance therapeutic angiogenesis (Rehman et al., 2004; Cao et al., 2005). Currently, most gene therapies for PVD are based on the use of viral vectors, which always raises safety concerns (Hacein-Bey-Abina et al., 2008; Keeney et al., 2013). In recent years, some studies have begun to focus on nanomaterials, which can act as non-viral gene delivery tools and enhance the transfection efficiency at the time (Green et al., 2008; Yang et al., 2009). One study demonstrated the use of NPs to transfect stem cells *in vitro* and suggested methods for validating the efficacy of using VEGF-expressing stem cells to promote angiogenesis in a murine ischemic hind limb model (Keeney et al., 2013). Another study indicated that CXCR4 is expressed on progenitor and inflammatory cells, facilitating cell migration toward ischemic tissues where they participate in revascularization and tissue repair. The result of this study showed that NP-induced CXCR4 overexpression may promote favorable phenotypic changes in and the therapeutic efficacy of stem cells in response to an ischemic environment in a murine model of peripheral arterial disease (Deveza et al., 2016). In addition, nanocapsules constitute a universal and highly efficient biomimetic platform for the transfer of genetic material. A recent study showed that calcium carbonate NPs, upon entering the MSCs, will modulate the local intracellular pH, leading to a delay in the degradation of the layers, and hampering the release of functional RNA molecules, which led to a decrease in delivery efficiency and indicated their higher stability (Tarakanchikova et al., 2020). Figure 2 shows the mechanisms of NPs combined with stem cells and applied for vascular regeneration.

## The Limitations and Future Direction of NPs for CVD Therapy

The main problem with NPs is their toxicity, including cellular toxicity and tissue toxicity. As the size of NPs decreases, the surface area increases, leading to a dose-dependent increase in the oxidation and DNA destruction capacity of the nanomaterials (Risom et al., 2005). NPs with a high aspect ratio have the greatest toxicity (Hsiao and Huang, 2011). Also, higher cationic charges cause severe toxicity because of hemolysis and platelet aggregation (Gatoo et al., 2014). Approaches such as arginine-glycine-aspartic acid peptide conjugation have been attempted. High-performance arginine-glycine-aspartic acid-conjugated dendrimer-modified gold nanorods exhibited great potential in applications, such as tumor targeting, imaging, and selective photothermal therapy (Li et al., 2010). In the future, these nanoprobes may be applied for use in CVD therapy.

Limited knowledge regarding the fate of these NPs and the extent of their accumulation in internal organs creates another bottleneck in the efforts for determining the potential side effect of their accumulation in the body. For example, when stem cells die, SPIONs remain in the body and produce images that misdirect the tracer and overestimate the extent of stem cell survival. QDs may impact the behavior of stem cells, leading to unexpected results. For example, stem cells labeled with QDs exhibited alterations and abnormality (Hsieh et al., 2006). Controlled and smart liberation of nanomaterials or their functional moieties and subsequent activation of the angiogenic signaling cascade in a spatiotemporal manner is exceptionally critical for positive therapeutic angiogenic outcomes.

Despite the various developments in tissue engineering, the challenges to developing fully functional vascularized tissue that recapitulates the complexities of the native tissue remain. The mechanisms of angiogenesis, the role of the scaffold architecture, and the interaction of potential biological and inorganic cues with the developing vasculature are among the key parameters to be considered as research progresses to a higher level. Efforts should be taken to enhance capillary network infiltration without compromising the physical and mechanical characteristics of the scaffolds (Patel and Lee, 2015). Besides, the potential accumulation of NPs in the liver and kidneys and whether such accumulation increases organ damage must be ascertained. In future studies, the mechanisms of stem cell-based cardiac and vascular regeneration need to be more comprehensively understood. Further studies are also needed for in-depth exploration of the possible effects of NPs parameters such as size, charge, morphology, surface characteristics (Carlander et al., 2018), as well as their absorption, distribution, and metabolic mechanisms *in vivo* (Carlander et al., 2016).

Compared with traditional treatments, NPs have shown potential superiority for use in therapy. Their particular chemical and physical advantages influence stem cell activity, such as through their use as non-viral gene delivery tools. As gene therapy for CVD has become increasingly popular, NPs applications are promising. In tumor therapy, smart micro/nanoparticles (MNPs) can react in a predictable and specific manner to external/internal stimuli. These MNPs including pH-sensitive peptides and polymers, redox-responsive micelles and nanogels, Thermo- and magnetic-responsive NPs, mechanical- and electrical-responsive MNPs, light- and ultrasound-sensitive particles. Multiresponsive MNPs include dual stimuli-sensitive nanosheets of graphene (Karimi et al., 2016), which have been rarely applied in stem cell-based CVD therapy.

## Conclusion

NPs might have significant potential uses in human treatments in the future, thereby meeting the changing needs of individual patients worldwide.

## Author Contributions

ZL provided the topic of this review and revised the manuscript. YS and YL wrote and contributed equally to the manuscript. LY helped in correcting the logic and some details of the manuscript. All the authors read and approved the final manuscript.

## Conflict of Interest

The authors declare that the research was conducted in the absence of any commercial or financial relationships that could be construed as a potential conflict of interest.
